# Fat necrosis: A neglected side effect of intramuscular injections

**DOI:** 10.1002/ccr3.5971

**Published:** 2022-06-19

**Authors:** Kouki Chaima, Amouri Mariem, Bahloul Emna, Charfi Slim, Hammemi Fatma, Boudawara Tahya, Turki Hamida

**Affiliations:** ^1^ Department of Dermatology Hedi Chaker Hospital Sfax Tunisia; ^2^ Department of anatomopathology Habib Bourguiba Hospital Sfax Tunisia

**Keywords:** fat necrosis, intramuscular injection, meglumine antimoniate

## Abstract

Panniculitis has various etiologies. One of the less common causes is trauma and hence traumatic fat necrosis (FN). These soft tissue injuries usually appear on the shins, thighs, breasts, arms, and buttocks. FN is mainly caused by trauma and may be associated with other conditions such as pancreatic disease. FN arising after intramuscular injections is uncommon and usually appears as firm, encapsulated, mobile, nontender, and solitary or multiple subcutaneous nodules. We report an interesting case of FN caused by intramuscular injections of cefazolin and meglumine antimoniate (MA) in a 38‐year‐old female patient. MA is regarded as the first‐line systemic treatment for cutaneous leishmaniasis (CL). However, these drugs are not devoid of various potentially adverse reactions.

## Graphical abstract

Intramuscular injection (IM) into the gluteal muscles remains a common route of medication but may lead to complications. Although rare, and with very few published similar case reports, it remains possible that FN resulting from meglumine antimoniate (MA) injections is underrecognized and underreported. It is important for healthcare professionals to be careful of fat necrosis as a possible side effect of MA injections and to elaborate adequate imaging investigations and surgical management in those patients with localized and persistent superficial nodular pain and tenderness at injection sites. We report an interesting case of subcutaneous FN caused by repetitive IM injection in a 38‐year‐old female patient referred to our department for the management of cutaneous leishmaniasis.

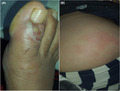

## 1 INTRODUCTION

Fat necrosis (FN) is a localized panniculitis. Most cases are caused by trauma, although some have been described following injection therapy. It is a benign nonsuppurative inflammatory disease of adipose tissue. It is a rare condition, affecting all ages.^1^ Terminology of FN remains confusing as it is also called “nodular‐cystic fat necrosis,” “mobile encapsulated lipoma,” “nodular fat necrosis,” and “post‐traumatic fat degeneration”.^1^, ^2^ FN is the consequence of the stimulation of an inflammatory reaction within the adipose tissue, which is due to a local trauma or tissue injury.^1^, ^3^

Herein, we present a distinctive case of FN occurring after intramuscular injection of meglumine antimoniate and cefazolin.

## 2 CASE REPORT

**FIGURE 1:**
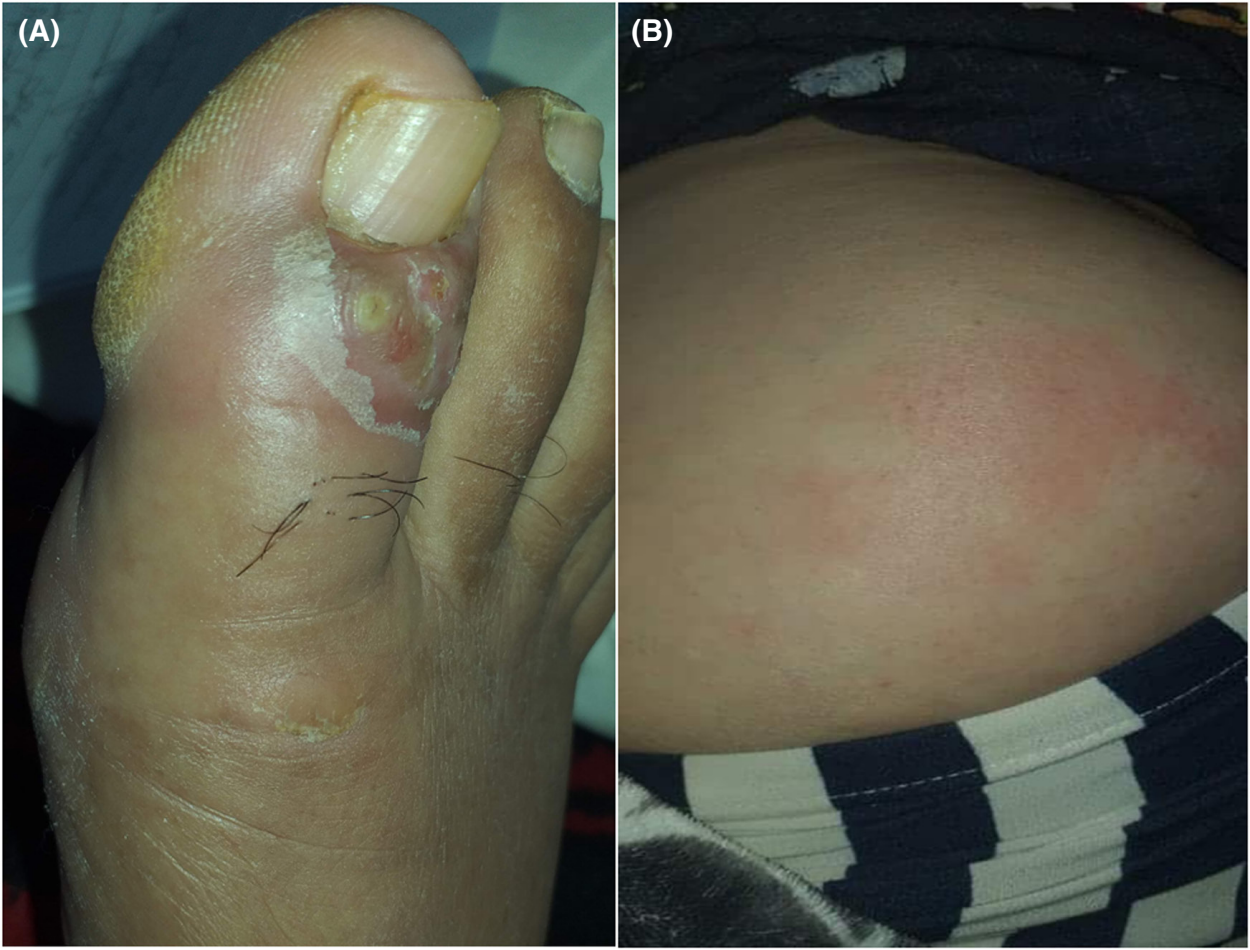
(A) Paronychia of the big toe. (B) Indurated plaque with red‐orange surface of the buttock

**FIGURE 2:**
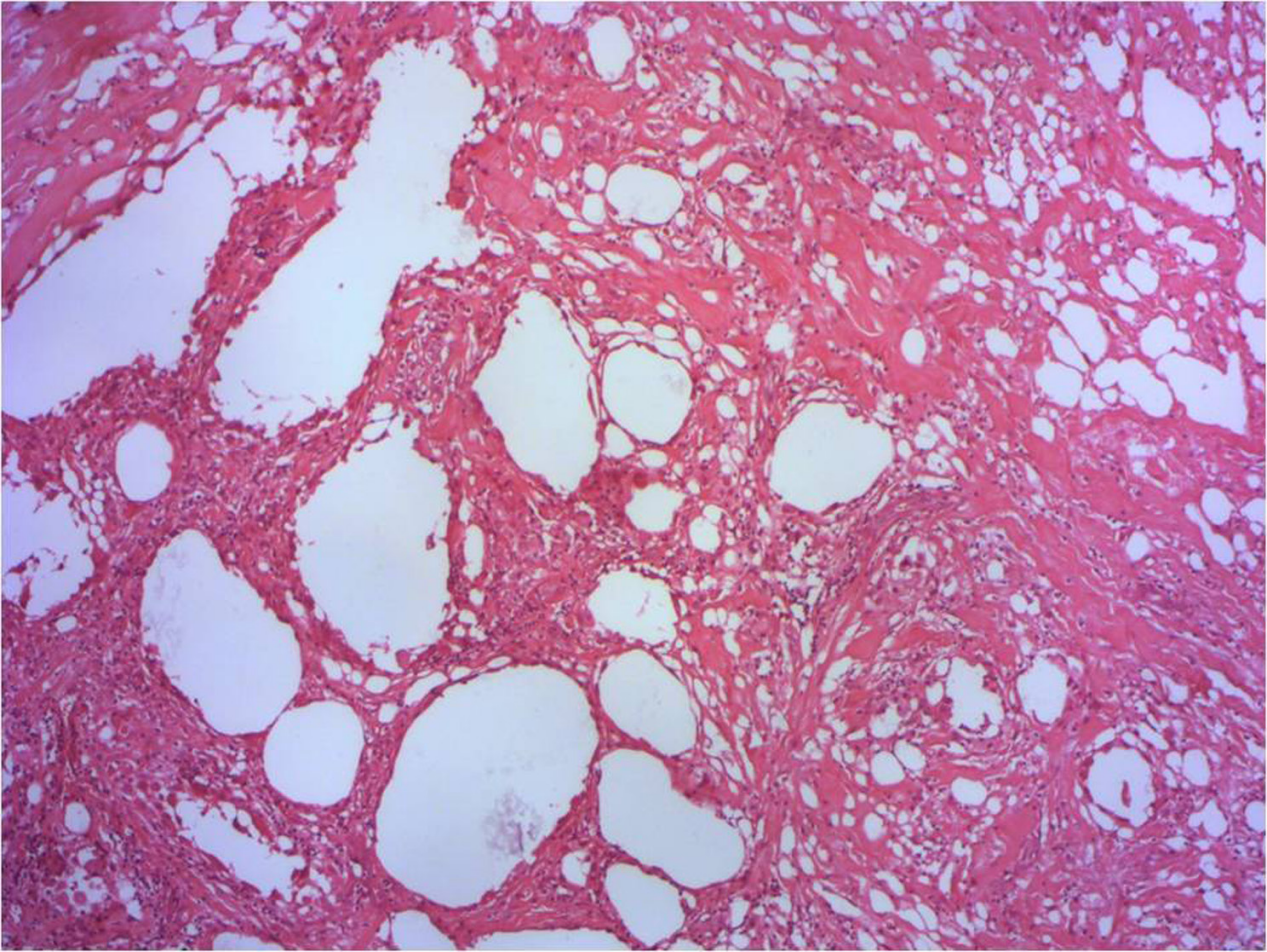
Extensive lesions of steatonecrosis. Note the cystic degeneration of adipocytes and the presence of numerous multinucleated foreign body type giant cells (HE × 50)

A 38‐year‐old woman was admitted to our department with asymptomatic multiple ulcerative nodules of 1‐month duration. Her medical history was unremarkable. Dermatological examination revealed an ulcerocrusted nodule on the right leg and the left forearm and multiple subcutaneous nodules extending along the line of lymphatic vessels. The patient also had a paronychia of the big toe (Figure 1A) and an indurated plaque of 70/40 mm on the right buttock (attributed to intramuscular injections of cefazolin 2 times per day for 1 week). The remaining physical examination was normal except for obesity (Body Mass Index of 30). The diagnosis of cutaneous leishmaniasis (CL) was confirmed by a positive polymerase chain reaction (PCR). Given the clinical form of CL (sporotrichoid, paronychia, and multiple lesions), intramuscular injection of meglumine antimoniate (IMMA) was initiated (at the dose of 30 mg/kg/day for 5 days and 60 mg/kg/day for 8 days). Despite the deep intramuscular injection afar from the indurated plaque of the buttock (Figure 1B), the patient remained annoyed, which compelled us to stop IMMA. An ultrasound of the right buttock plaque showed a large hypoechogenic mass on the deep tissue away from the muscle without any detectable collection. We recommended a high‐potent topical corticosteroid (3 months) with no significant improvement. We associated an oral colchicine (1 mg/day) for 9 months. Thereby, a limited excision was made 27 months after the onset of the lesion of the buttock. The histology showed extensive lesions of steatonecrosis with a cystic degeneration of adipocytes and numerous multinucleated foreign body type giant cells (Figure 2). The diagnosis of fat necrosis (FN) induced by intramuscular injection of an antibiotic and worsened by repetitive injections of IMMA was made. No recurrence was observed during 6 months of follow‐up.

## 3 DISCUSSION

First described on the breast in 1920, FN is a benign nonsuppurative inflammatory disease of adipose tissue. It is an uncommon, self‐limited panniculitis of traumatic origin in newborns, while it mainly affects women after a breast surgery.^1^ Limbs, which are commonly exposed to repeated microtrauma, are the most commonly affected sites. Numerous diseases have been reported in association with FN: pancreatitis, collagen vascular diseases, myeloproliferative disorders, asphyxia, hypothermia, subcutaneous injections, and trauma.^3^, ^4^

In fact, the fat is composed of microlobules of fat cells; each microlobule is serviced by a blood vessel. In situations where there is pressure on the fat compartments, they burst and the surrounding septa and vessels rupture, leading to the damage of the fat cells. The damaged fat cells are transformed into glycerol and fatty acids.^2^ They are responsible of a continuous irritation and aggravate the initial lesions. Obesity, the only morbidity found in our patient is more likely to be a risk factor. We generated this hypothesis with the belief that the subcutaneous adipose tissue of obese patients has a lower blood flow.

Clinically, FN presents as indurate painful deep nodules of the subcutaneous tissue covered by a normal skin or an “orange‐peel” appearance, local depression, or discoloration of the skin.^1^ The acute presentation as a large indurated plaque, as in our patient, is quite unusual.^4^

FN has a similar imaging appearance as mature adipose tissue. Ultrasonographic image displays a hypoechoic appearance in most cases (like ours). However, FN of the trunk and in extremities are not fully characterized.^5^

Intramuscular injection (IM) into the gluteal muscles represents a common route of medication, and it may lead to various complications. A retrospective study including 32 patients who required surgical treatment for local complications of buttock injections in children was made at the Taegu Fatima Hospital during a 7‐year 9‐month period. They observed local complications such as acute inflammation, cellulitis and abscess (71.9%), FN (21.9%), and injection granuloma (6.2%).^6^

Chemical agents such as vasopressin, human growth hormone, triamcinolone, and insulin are also mostly reported.^3^, ^4^

In our patient, FN was induced by IM injections of cefazolin and compounded by IMMA. Several cutaneous side effects of these molecules were previously reported. First, MA is known for its different adverse drug effects: either general reactions (including rash and urticaria) or local ones (infection, rash, etc.). According to the large series of Beheshti et al.,^7^ the most common side effects of MA are skin hypersensitivity and urticaria. Most authors explain this high frequency by the presence of impure heavy metals in the product.^8^ Second, cefazolin is an antibiotic known for its allergic reactions and cross‐reactivity with beta‐lactams.^9^ Hypersensitivity to cefazolin is well documented, and the symptoms usually include urticaria, diarrhea, vomiting, and transient neutropenia, which are rarely life‐threatening.^10^ Local cutaneous side effects aren't reported in the literature. To the best of our knowledge, there was no previous case of FN induced by intramuscular injection of this antibiotic.

The diagnosis of traumatic panniculitis was first proposed in our patient, hence the prescription of colchicine. Given the resistance, a cutaneous calcinosis or foreign body granuloma was suspected. An excision confirmed the diagnosis of FN. To the best of our knowledge, there were no previous case reports of FN post‐cutaneous leishmaniasis treatment.

Histopathology findings in FN are polymorph and depend on the stage of the disease. At early stages, FN with cystic spaces and numerous neutrophils in the adjacent fat is seen. Later on, predominant cells become lymphocytes, histiocytes, and multinucleated giant cells with lipid vacuoles in some cells. Finally, fibrosis prevails in histology.^2^

Since FN is a self‐limiting condition, a conservative approach can be followed. Surgical intervention is required only in patients with intractable pain and complications^2^, ^3^ or for cosmetic reasons. In our patient, we performed a surgical excision given the impact of the lesion and the unusually persistent character.

## 4 CONCLUSION

We report an original case of FN. It is the first one described after IM cefazolin injections and compounded by repetitive IMMA. We believe that the injections on the superficial fat tissue in an obese patient compromised local vascularization and caused a FN with a cystic degeneration and a foreign body reaction. Although cutaneous leishmaniasis is a benign parasitic infection, lesions can lead to disability and disfiguring scars. Systemic treatment remains the main treatment in these cases. Follow‐up and early detection of adverse drug reaction of IMG is of a good help for better management.

## AUTHOR CONTRIBUTIONS

Dr Kouki Chaima wrote the manuscript and is the guarantor of the content of the manuscript, including the data and analysis. Dr Amouri Mariem analyzed, interpreted, and critically revised the data. Dr Hammami Fatma and Bahloul Emna contributed to interpretation of data and revision of the manuscript. Dr Slim Charfi provided the anatomopathological figures. Dr Hamida Tuki and Dr Boudawara Tahya contributed to the final approval of the version of the manuscript to be submitted.

## CONFLICT OF INTEREST

None.

## ETHICAL APPROVAL

The ethics statement was approved.

## CONSENT

The consent statement was approved by all authors. Written informed consent was also obtained from the patient to publish this report in accordance with the journal's “patient consent policy.”

## Data Availability

None
